# Needle aspiration technique as a supportive tool for clinical diagnosis of anorectal atresia in calves

**DOI:** 10.1186/s12917-025-04912-8

**Published:** 2025-07-15

**Authors:** Ahmed Abdelrahiem Sadek, Kamal Hussein

**Affiliations:** Department of Surgery, Anesthesiology, and Radiology, College of Veterinary Medicine, Assiut University, Assiut, 71526 Egypt

**Keywords:** Atresia Ani, Atresia Ani et recti, Needle aspiration, Congenital anomalies, Calves

## Abstract

**Background:**

Early diagnosis and treatment of anorectal atresia, a common congenital abnormality in calves, are crucial for preventing complications and ensuring the animal’s survival. Typically, newly born calves with this condition are present with an inability to defecate due to an absence of or an obstructed anal opening, often accompanied by abdominal pain and distension. History, physical examination, and radiographic imaging are frequently utilized diagnostic tools. This study aimed to evaluate the effectiveness of needle aspiration as a supportive diagnostic technique for anorectal atresia in bovine calves under field conditions and to assess its role in decision-making to proceed with surgical intervention.

**Results:**

A total of 116 male calves, aged six hours to five days, were examined through clinical inspection, needle aspiration, and plain radiography. Clinical findings indicated that 62 cases had atresia ani, while 54 calves were diagnosed with atresia ani et recti. In cases without detectable swelling under the base of the tail, even with manually applied pressure on the abdomen, needle aspiration and radiographic findings showed positive results in 46.30% of calves. These cases were characterized by a radiolucent, distended rectal end close to the perineal skin surface (≤ 5 cm). Conversely, 53.70% of animals had negative aspiration results, with radiographic evidence of gas accumulation at the rectal end located > 5 cm from the perineal surface. Additionally, successful creation of an artificial anus at the perineum was achieved in cases with a rectal end near the skin surface. Whereas cases with a far rectal end more than 5 cm were subjected successfully to right flank laparo-typhlostomy.

**Conclusions:**

Needle aspiration is a straightforward, non-invasive technique that proves highly valuable in facilitating diagnosis and guiding surgical decisions in calves with anorectal atresia, particularly in cases where bulging is not observed upon manual abdominal pressure. It is most effective when the rectal end is within five centimeters proximal to the perineal skin surface.

## Background

Congenital disorders in newborn bovine calves have become increasingly prevalent in recent decades due to factors such as genetic mutations and environmental issues [1, 2]. Among these various anomalies, intestinal abnormalities are the most common reported disorders in newly born calves, with anorectal anomalies particularly atresia of the anus and/or rectum being the most frequently encountered malformations [1–3].

Anal atresia is characterized by the congenital absence of the anal opening, whereas rectal atresia refers to the absence of a segment of the rectum. Atresia ani and atresia recti may occur independently or in combination [1, 2, 4]. Anorectal atresia can be classified into four types based on the affected portion: Type I is characterized by a stenotic anus with a normal rectum. Types II and III involve the imperforation of the anus with the rectal termination forming a blind pouch, either immediately behind the imperforate anus (Type II) or more cranially within the pelvis (Type III). Type IV represents the absence of the cranial portion of the rectum in the pelvic cavity while the anal opening and terminal portion of the rectum remain intact [5–7].

Anorectal atresia is a life-threatening condition to calves due to the time-sensitive onset of endotoxemia, dehydration, and peritonitis, resulting from fecal accumulation or bowel rupture. Therefore, early and accurate diagnosis and management are crucial for increasing the survival rate of the affected calves [3, 6, 8].

Diagnosis of atresia of anus and/or rectum mainly depends on the animal’s history and clinical presentation. Affected calves are often admitted within the first few hours after birth, exhibiting symptoms such as failure to defecate, abdominal distension, tenesmus, and anorexia [1, 9]. Additionally, calves with Type II atresia ani present an imperforate anus with a visible mild soft swelling in the perineal region beneath the base of the tail, while those with Type III atresia ani et recti lack an anal orifice and visible bulging, even when manual pressure is applied to the abdomen [1, 6, 10, 11]. Radiography also plays a crucial role in the definitive diagnosis of anal and rectal atresia, aiding in differentiating between the types of anorectal atresia [1, 12]. Plain radiographs reveal radiolucent zone of gases occupying the various parts of the gastrointestinal tract, depending on the type of atresia. In cases of atresia ani, the radiolucent air-filled structure is observed just cranial to the perineal skin surface below the base of the tail, while in atresia ani et recti, a gas-filled distended region is visible further cranially within the pelvic cavity [1, 3, 12, 13].

Needle aspiration, a minimally invasive and practical procedure, is widely used in veterinary practice for collecting samples from organs or lesions. This technique assists in confirming the diagnosis of various conditions, including abscesses, bursitis, hematomas, urethral dilation, and tumors [2, 14–16]. Additionally, needle aspiration provides a safe, simple, rapid, and cost-effective procedure with minimal complexity [16]. Consequently, this study hypothesized that the blind needle aspiration technique, applied to the perineal region under the tail base following an appropriate landmark, may facilitate the diagnosis of anorectal atresia in calves by evaluating the proximity of the blind rectal pouch to the skin based on the nature of the aspirated material.

Management of the anorectal atresia involves surgically creating a passage to allow for the evacuation of intestinal contents. Correction of atresia ani involves reconstructing the anal opening at the perineal region by fixing the blind rectal end to the skin, whereas the management of atresia ani et recti may involve laparo-colostomy or laparo-typhlostomy [1–3, 10].

The primary objective of this study is to describe the findings of needle aspiration, a less invasive technique, in cases of anal and/or rectal atresia. The study also aims to correlate these findings with the results of clinical and radiographic examinations of the same cases. Furthermore, it seeks to differentiate between anal and rectal atresia based on the needle aspiration technique, thereby aiding in the definitive diagnosis and guiding subsequent surgical management.

## Materials and methods

The study protocol was approved by the Ethical Committee of the Faculty of Veterinary Medicine, Assiut University, Assiut, Egypt, according to the standards of Office International des Epizooties (OIE) and Egyptian laws for the use of animals in research and education (No. 06/2024/0265). All procedures in the current study were conducted in compliance with the guidelines of Animal Research Reporting of In Vivo Experiments (ARRIVE). Informed consents for the use of animals were obtained from the animal owners.

### Animals

This study included 116 male bovine calves (72 native Baladi breed and 44 cross breed), aged between six hours to five days, and weighing 45–60 Kg, presented to the Veterinary Teaching Hospital, Faculty of Veterinary Medicine, Assiut University, Egypt, between January 2018 and August 2024.

### History and clinical examination

Calves were admitted with the primary complaint of an obscured anal opening, lack of defecation, and signs of abdominal discomfort since birth. A thorough clinical examination was conducted, evaluating presence or absence of perineal bulging under manual abdominal pressure, abdominal distension, signs of abdominal pain, and the overall health condition of the calves.

### Needle aspiration method

Blind needle aspiration was performed after surface anesthesia using 2% lidocaine gel (Xylocaine^®^, AstraZeneca Co., Egypt) under aseptic conditions. A sterile 18G needle with a 20-mL syringe (21.55 mm diameter; ELDAWLIA Med Co., Egypt) was inserted just beneath the base of the tail, directed perpendicularly to the perineum and parallel to both the sacrum and tail base, to safely aspirate rectal contents and blindly assess the proximity of the atretic rectum to the skin.

### Radiological assessment

All calves underwent plain radiography of the caudal abdomen to confirm and differentiate between atresia ani and atresia ani et recti. Lateral radiographs were obtained using a fixed X-ray apparatus (DrGEM GXR-SD, South Korea) and a computed radiograph system (Fuji Film FCR PRIMA T2, Japan) with exposure settings of 45–50 kV, 15 mA/s, and a focal film distance (FFD) of 75–80 cm.

### Surgical management

Management of the presented cases was carried out by either creating an anal opening at the perineal region under the base of the tail or performing a right flank laparo-typhlostomy.

Creation of anal opening at the perineal region under the tail base was performed as described previously [2]. The perineal area was aseptically prepared, followed by local infiltration with 0.5% lidocaine HCl (Debocaine^®^, El-Nasr Pharm Chemicals Co., Egypt). Following adequate desensitization of the surgical site, a circular skin excision was done, and blunt dissection was performed until the blind end of the rectum was reached. Then, the rectal end was gently grasped, fixed to the skin with simple interrupted sutures using silk (M-Natur^®^, International Sutures Manufacturing Co., Egypt), and the blind end was opened.

The right flank laparo-typhlostomy was conducted according to MAH Abdel-Hakiem and NM Aref [1]. In brief, calves were positioned in left lateral recumbency, restrained appropriately, and the right flank region was prepared aseptically. A linear infiltration anesthesia of 0.5% lidocaine HCl was administrated adequately, followed by a linear incision through the abdominal wall to explore the abdominal contents and locate the blind-sac cecum. The cecum was sutured to the abdominal muscles with polyglycolic acid (M-Natur^®^; International Sutures Manufacturing Co. Egypt) using simple continuous sutures. At last, the blind sac was opened, and the cecal edges were sutured to the skin using a silk (M-Natur^®^, International Sutures Manufacturing Co., Egypt) in a simple interrupted suture pattern.

## Results

### History and clinical findings

**Fig. 1:**
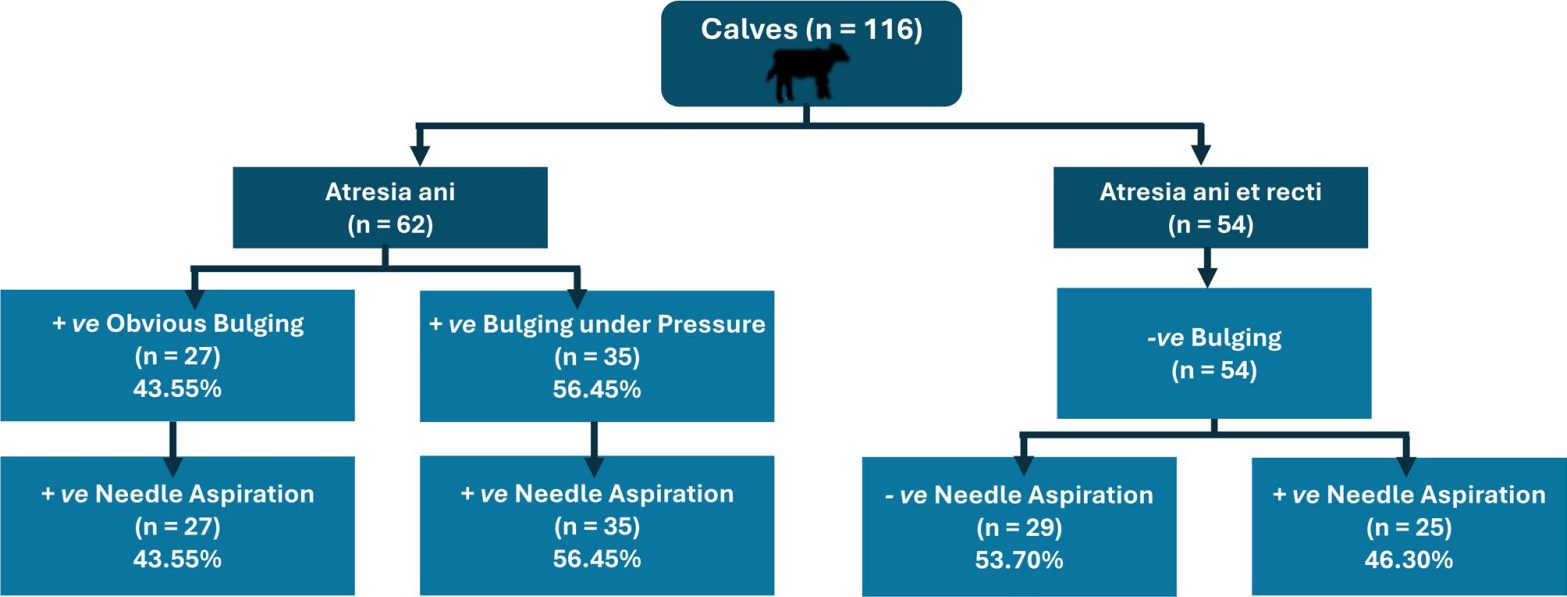
Schematic flowchart of calves with atresia ani and atresia ani et recti, classified based on clinical bulging and needle aspiration findings

The calves were depressed, mildly dehydrated, and exhibited signs of abdominal distention and tenesmus. On examination, 62 calves (53.45%) showed a bulging under the tail base at the perineal region, suggesting anal atresia (Fig. 1). Among these, 27 calves (43.55%) had obvious bulging, while 35 (56.45%) only displayed bulging with digital pressure applied to the flank region. In contrast, 54 calves (46.55%) exhibited no bulging at the perineum even under manual abdominal pressure, indicating atresia ani et recti.

### Needle aspiration outcome

**Fig. 2:**
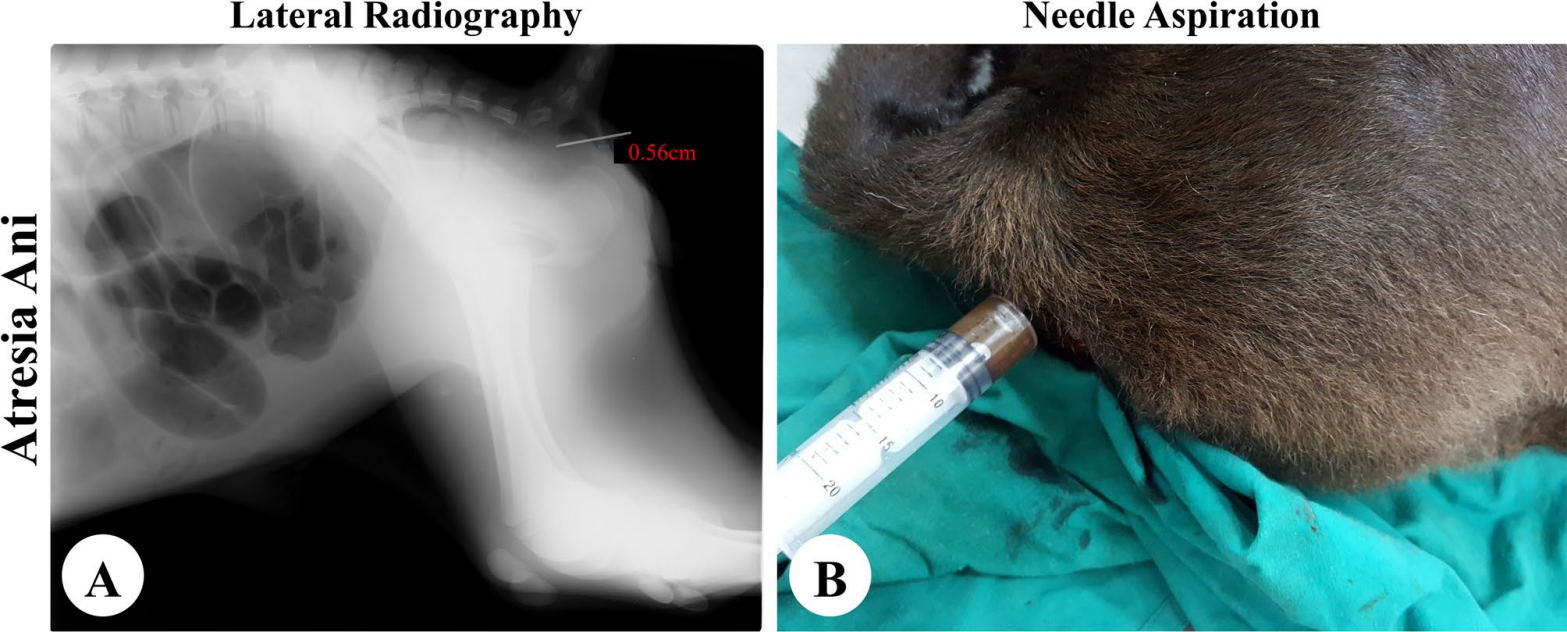
The findings of atresia ani on plain radiography (**A**) and needle aspiration (**B**)

**Fig. 3:**
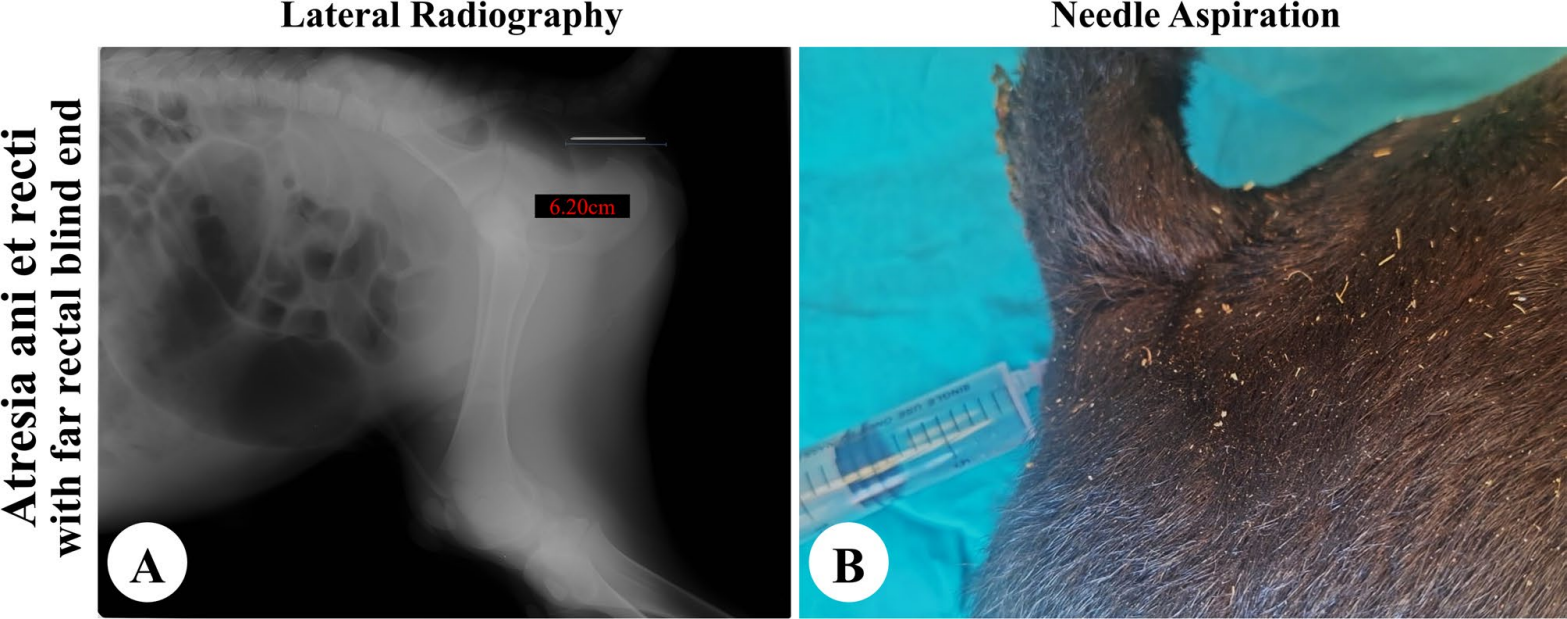
The findings of atresia ani et recti with a far rectal end (> 5 from skin surface) on plain radiography (**A**) and needle aspiration (**B**)

**Fig. 4:**
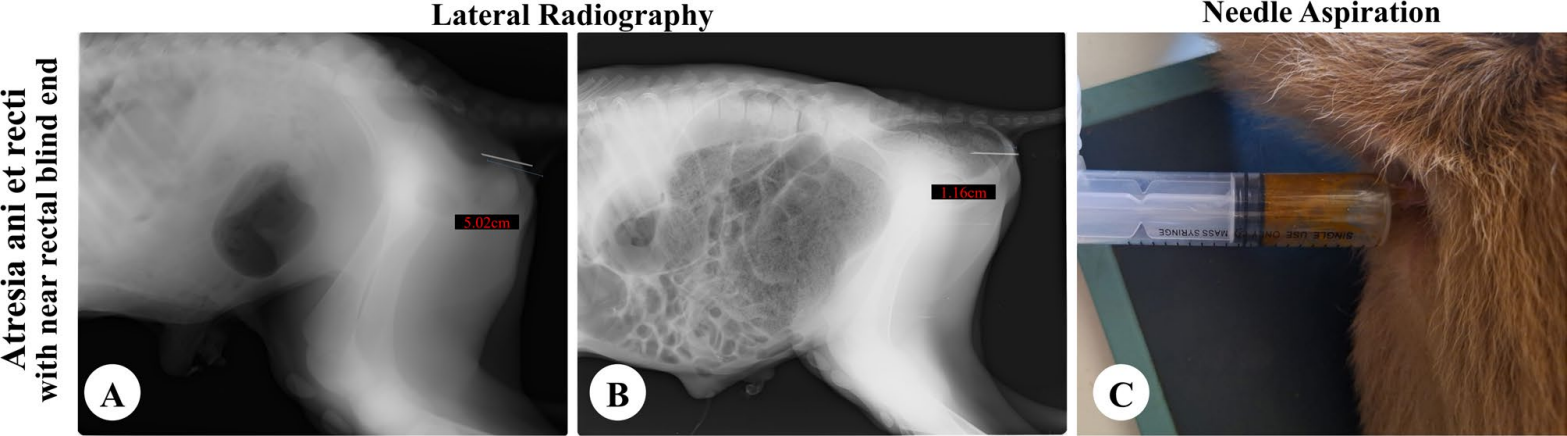
The findings of radiography (**A** and **B**) and needle aspiration (**C**) of atresia ani et recti with a rectal end ≤ 5 cm from the surface of perineal skin

Needle aspiration in calves presented with either obvious bulging (*n* = 27) or manual pressure-induced bulging (*n* = 35) revealed a positive fecal aspiration, confirming anal atresia (Fig. 2B). Of the 54 calves without bulging, aspiration was negative in 29 cases (53.70%), suggesting anorectal atresia with a distant rectal end (more than 5 cm from the skin surface) (Fig. 3B). Positive aspiration was observed in 25 cases (46.30%), indicating atresia ani et recti with the rectal end near the perineal surface (within the needle’s reach) (Fig. 4C).

### Radiographic assessment

In the 62 calves diagnosed with atresia ani with obvious bulging under the tail base or bulging under abdominal pressure manually, lateral radiographs revealed a radiolucent gas-filled area extending from the pelvis to just cranial to the obscured anus (Fig. 2A).

In cases of atresia ani et recti that were admitted without bulging even upon digital abdominal pressure, a distended area of gases at the blind rectum was located within the pelvis with more than 5 cm from the perineal skin surface in 29 calves as shown in Fig. 3A. The remaining 25 cases displayed a gas-filled area at the blind rectal end within the pelvic cavity with a distance of 1.16 to 5.02 cm from the skin surface (Fig. 4A, B). The radiopaque needle was visible inside the gas-filled rectal area in cases of anal atresia (Fig. 2A) and atresia ani et recti when the rectal end was located within 5 cm of the perineal surface (Fig. 4A, B). However, the needle was located outside the radiolucent gas-filled region in case of atresia ani et recti with a more distant rectal end from the body surface (Fig. 3A).

### Surgical findings

**Fig. 5:**
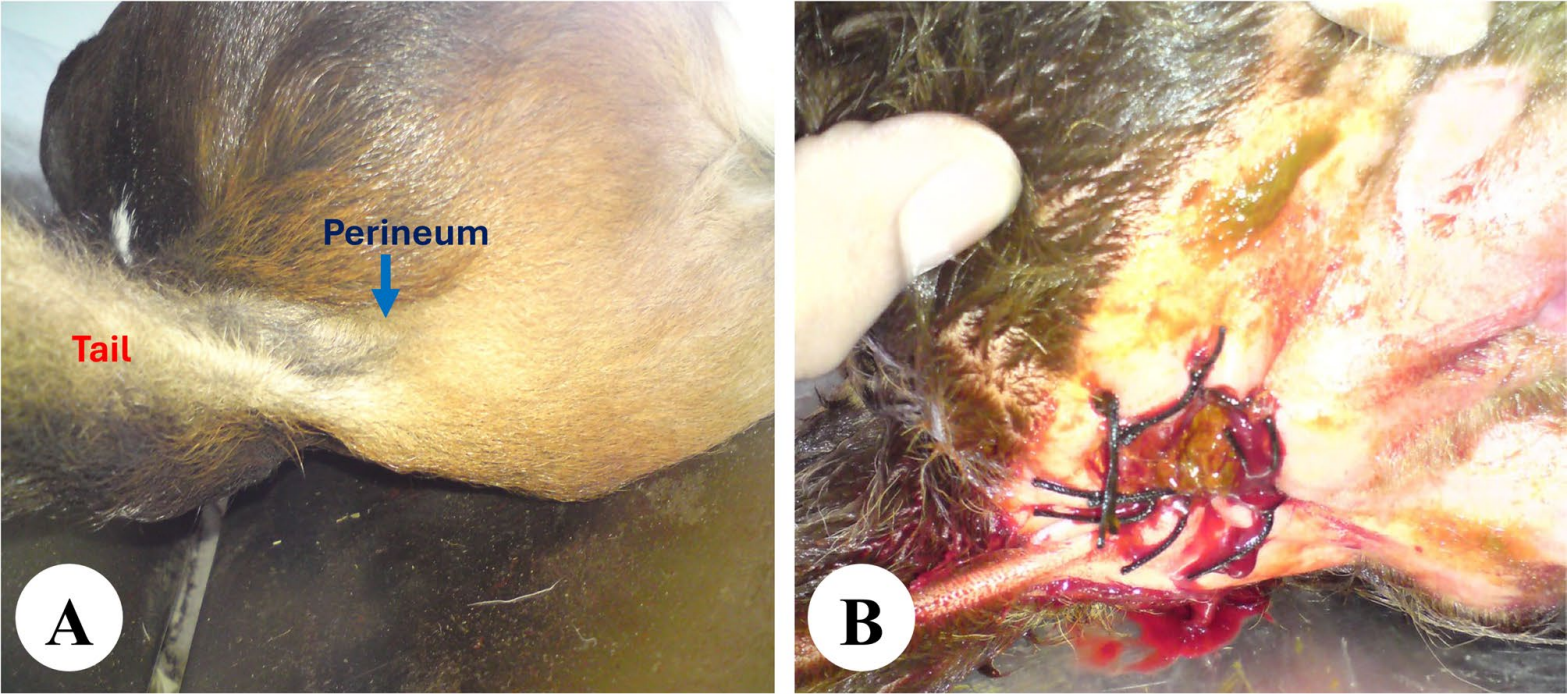
Surgical creation of anal opening at the perineum under the tail base. The operative site at the perineum under tail base (**A**). The rectal end is exteriorized and sutured to the skin (**B**)

**Fig. 6:**
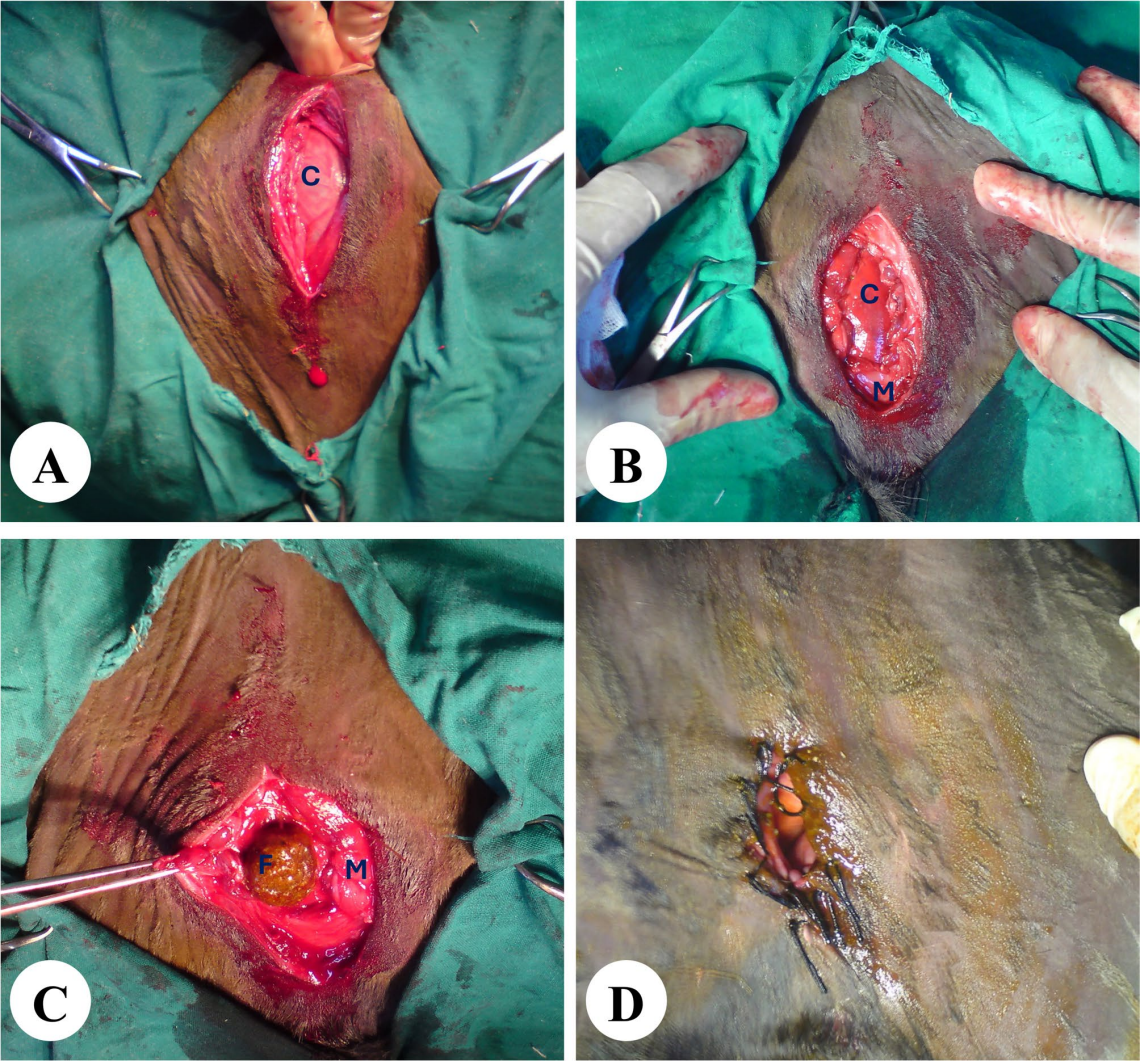
Laparo-typhlostomy procedure. Exposure of the cecum (**A**) followed by fixation to the abdominal wall muscles (**B**). Then, opening of the cecum (**C**) and suturing the cecal wound to the skin (**D**). C: cecum; F: cecal contents; M: abdominal muscles

Creation of anal opening at the perineal region under the base of the tail (Fig. 5A, B) was performed for treatment of calves with atresia ani and those with atresia ani et recti when the rectal end was within 5 cm of the skin surface and positive needle aspiration results. This method successfully allowed the blind rectal end to be grasped and fixed. Calves with atresia ani et recti and a rectal end more than 5 cm from the perineal surface, as indicated by radiographs and negative needle aspiration, were managed effectively with right flank laparo-typhlostomy (Fig. 6A-D).

## Discussion

This study evaluates needle aspiration as a supportive diagnostic tool for anorectal atresia in calves, correlating it with clinical signs, radiographic findings, and surgical outcomes. A total of 116 calves with suspected anorectal atresia were included, and the study demonstrated the utility of needle aspiration for distinguishing between atresia ani and atresia ani et recti, particularly when the rectal sac is close to the skin surface. Radiographic imaging confirmed the diagnosis, consistent with the finding of initial needle aspiration, which further assisted in determining the correct surgical approach (i.e., creating a rectal opening at the perineal region below the tail base or right flank laparo-typhlostomy).

In calves without clear perineal bulging, determining whether the rectal sac is near to the skin can be challenging [2, 11, 17]. However, the needle aspiration technique used in this study proved valuable in distinguishing cases where the rectal blind end was close to the skin, providing crucial information for surgical decision-making. Positive aspiration of fecal matter in 46.30% of cases with no bulging suggested that the rectal end was close enough to the skin to allow for effective creation of an anal opening. This surgical procedure is considered a simple, quick, and successful technique with low complexity. It can be performed in the field, offering a good prognosis with minimal complications [6, 9]. However, several complications of the needle aspiration technique have been reported, including bruising, swelling at the aspiration site, bleeding or hematoma, pain, damage to surrounding tissues, nerve injury, and needle track sinus formation [18–20]. Notably, none of these complications were observed in the present study. Moreover, the efficacy of needle aspiration can be limited by factors such as the surgeon’s expertise, the consistency of rectal contents (especially in cases with dried or semi-solid fecal matter), and the presence of concurrent congenital abnormalities affecting the perineum, pelvis, or hindquarters.

Conversely, negative aspiration indicated a more distant rectal blind end (over 5 cm from the perineal surface), requiring a different surgical approach, such as right flank or ventral laparotomy with typhlostomy or colostomy [2, 21]. These interventions provide a safe method for evacuating gastrointestinal contents, thereby preserving life and extending survival from two to six months [1–3, 9, 21, 22]. Despite their benefits, these approaches carry risks, including surgical site infections, stenosis or obstruction of the fistula, leakage of intestinal contents, and subsequent peritonitis or sepsis [9, 21]. Long-term functionality may be affected by mucosal prolapse, necrosis, or poor wound healing, especially in fragile or compromised animals [21, 22]. In this study, all operated calves survived during the three-month follow-up until slaughter. However, skin scalds distal to the abdominal wall wound were observed in 27 calves, and cecal wall hypertrophy was noted in all animals after 30–35 days post-surgery.

The importance of needle aspiration in diagnosing anorectal atresia lies in its ability to provide a quick, minimally invasive method to determine whether the rectal sac is near the perineal skin. This is particularly useful when radiography alone does not provide sufficient information, and if radiography facilities are unavailable, especially under field conditions. In cases of atresia ani et recti without perineal bulging, needle aspiration allows veterinarians under field conditions to assess whether the rectal blind end can be surgically manipulated through the perineum or if a more invasive approach, like laparotomy, is necessary. Thus, this technique, combined with radiographic imaging, improves the accuracy of the diagnosis, helping veterinarians make informed decisions about the most effective surgical procedure.

The use of plain radiographs complements needle aspiration by showing the location of gas-distended sections of the rectum, thus providing a non-invasive way to determine the approximate location of the rectal end in relation to the perineal surface [3, 12]. Together, these two diagnostic tools greatly enhance the ability to distinguish between different forms of anorectal atresia, particularly in challenging cases where the rectal end is not easily accessible via physical examination alone [23]. Besides, various diagnostic imaging techniques including contrast radiography, ultrasonography, and endoscopy have been used to diagnose intestinal atresia, especially atresia coli, and to accurately localize the atretic segment [12, 24–26].

Only male calves were included in the current study, primarily due to clinical presentation patterns. Most cases of anorectal atresia presented at our facility involved male calves within the first week of life, as fecal retention leads to early onset of clinical signs. Based on our clinical observation, this condition remains fatal in males if not promptly treated, due to the progressive accumulation of feces. In contrast, female calves often present later (around two to three weeks of age), typically with a rectovaginal fistula that permits partial fecal evacuation and delays detection. Hence, the condition may be less acutely critical in females, as the presence of a rectovaginal fistula can provide an alternative route for fecal passage. This variation in clinical urgency likely accounts for the higher rate of early presentation in newborn males, as observed in the present study. Nevertheless, the described needle aspiration technique may also prove effective in female calves with anorectal atresia, warranting further investigation.

In conclusion, the integration of needle aspiration as an auxiliary diagnostic tool appears to facilitate and streamline the diagnostic process for anorectal atresia in calves. This approach offers an efficient, accurate, and minimally invasive method that supports determining the nature and extent of the anomaly and helps guide surgical decision-making.

## Data Availability

The data that support the results of this study are available from the corresponding author on reasonable request.
